# Deterioration and Oxidation Characteristics of Black Shale under Immersion and Its Impact on the Strength of Concrete

**DOI:** 10.3390/ma13112515

**Published:** 2020-05-31

**Authors:** Xin Liao, Wenda Zhang, Jiannan Chen, Qingfeng Wang, Xiyong Wu, Sixiang Ling, Deping Guo

**Affiliations:** 1 Faculty of Geoscience and Environmental Engineering, Southwest Jiaotong University, Chengdu 611756, China; xinliao@swjtu.edu.cn (X.L.); zhangwenda@my.swjtu.edu.cn (W.Z.); wuxiyong@swjtu.edu.cn (X.W.); lingsx@swjtu.edu.cn (S.L.); 2 MOE Key Laboratory of High-Speed Railway Engineering, Southwest Jiaotong University, Chengdu 611756, China; 3 World Heritage Research Center, Southwest Jiaotong University, Chengdu 611756, China; 4 Department of Civil, Environmental, and Construction Engineering, University of Central Florida, Orlando, FL 32816, USA; 5 Powerchina Raliway Construction Co., Ltd., Beijing 100048, China; qingfengwang@my.swjtu.edu.cn; 6 Sichuan Railway Investment Group Co., Ltd., Chengdu 610081, China; guodeping@my.swjtu.edu.cn; 7 School of Civil Engineering, Beijing Jiaotong University, Beijing 100044, China

**Keywords:** black shale, water-rock interaction, concrete, compressive strength, ultrasonic velocity

## Abstract

Black shale, which usually contains pyrite, is easily oxidized and generates acid discharge. This acidic environment is not favorable for concrete in engineering applications and is likely to affect the durability of engineering structures. This study investigated the effect of acid discharge from the weathering of black shale on the strength of concrete under partially immersed conditions. Black shale concrete immersion tests were conducted at different immersion depths to evaluate the oxidation conduction of black shale. Water chemistry and oxidation products were monitored during and after the immersion tests. The quality and strength of the black shale and concrete specimens were obtained before and after the immersion by testing the ultrasonic wave velocity and uniaxial compressive strength. The results indicated that a lower immersion depth of black shale reveals a higher degree of oxidation, and the capillary zone in black shale is critical for black shale oxidation in terms of mass transfer. The ultrasonic velocity of the concrete showed different change patterns in the immersed and non-immersed zones. Precipitation and additional hydration enhanced the quality and entirety of the concrete (increased ultrasonic velocity) at the non-immersed or partially-immersed zones, while the dissolution of concrete was dominant in the immersed zone (decreased ultrasonic velocity) and induced a reduction of concrete quality. The compressive strength of the concrete was enhanced after immersion. The concrete strength slightly increased by 5–15%. This phenomenon is attributed to the filling of the voids by the precipitations of minerals, such as goethite and anhydrite.

## 1. Introduction

Sulphide minerals are easily oxidized in an aerobic environment and generate acidic water in pores or fissures [1,2,3,4,5]. Black shale is a common sink of sulphide minerals, such as pyrite (FeS), and is susceptible to chemical weathering [6,7]. These processes include dissolution, precipitation, hydrolysis, hydration, and oxidation of the minerals, in which the oxidation of the pyrite in the black shale by dissolved oxygen in the pore water to generate ferric ions (Fe^3+^) and sulfate (SO_4_^2−^) is the most important chemical reaction [7,8,9,10]. The low pH/high sulphate drainage mentioned above, often referred to as acid rock drainage due to the oxidation of sulphide minerals, has gained attention in the past decades due to its potential impact on engineering structures and the environment [3,4,5,11]. The processes involved in the pyrite oxidation have been highly concerning as they lead to a number of engineering problems for the pyritic rocks, such as expansion, shear strength reduction, and acid corrosion [12].

The acidic water can have a strongly negative impact on the physical and mechanical properties of the surrounding rock masses (e.g., limestone and dolomite) or concrete structures (such as abutment) [2,3,4,5]. External sulphate attack on the concrete leads to the formation of gypsum, ettringite, and/or thaumasite, resulting in cracking, spalling, softening, expansion, loss of strength, and other forms of damage [6,7,8,9,10,11,12,13,14,15,16,17,18,19]. Rodrigues et al. [6] conducted mineralogical and chemical assessments of concrete damaged due to the oxidation of sulphide-bearing aggregates. They found that pyrrhotite (FeS, a nonstoichiometric variant of pyrite) oxidizes to form sulphuric acid and iron oxyhydroxides. The acid water then reacts with the cement paste/aggregate and forms sulphate minerals. The rate of corrosion of the concrete was further proven by Mahmoodian and Alani [13] to have a high correlation with the acidity of the water. Zhong and Wille [14] studied the effect of pyrrhotite-bearing aggregate on the deterioration of residential concrete foundations. They found that pyrrhotite and its oxidation products affect the surface of the aggregates of the deteriorated concrete. The abundance and spatial distribution of these secondary mineral formations are closely associated with concrete cracks due to their swelling. Schmidt et al. [15] evaluated the degradation of iron sulphide in concrete using physical and microstructural analysis. They found that the FeS and FeS_2_ present in the concrete showed a similar reaction behavior in the aggregates, and the expansion of the concrete is due to the formation of secondary ettringite caused by the released sulphate from iron sulphide. Park et al. [16] found that the sulphate attack could deteriorate the durability of high-strength concrete due to the expansion of the sulphate minerals, such as gypsum (CaSO_4_·2H_2_O). Similar observations were reported by Menéndez et al. [17], who performed immersion and semi-immersion tests to evaluate the resistance of sulfate-resisting concretes to external sulfate attack. Additionally, the acid water generated by the water-rock interaction of black shale could decompose the bonding materials in the concrete, such as calcium hydroxide (Ca(OH)_2_) and calcium silicate hydrate (CSH) gel [20].

The chemical kinetics of the water interactions of black shale are complex processes compared to those of pure sulphide minerals [7]. Currently, there is a limited number of studies that evaluate the oxidation of black shale and the effect of its oxidation product concurrently. Additionally, the aforementioned studies mainly focus on the influence of sulfate concentration and erosion time on the strength and structure of concrete through the full-immersion experiment. However, in field conditions, black shale is usually exposed to the atmosphere or partially submerged in water. The corresponding oxidation process may have different mechanics than under saturated conditions due to the mass transfer in the capillary zone, and limited studies have addressed these conditions.

In this study, the influence of acid water (sulfate-rich) generated by black shale weathering on the strength of concrete was evaluated by a specially designed immersion test setup. The weathering of black shale through water-rock interactions was assessed under different immersion conditions (partially immersed) in water. The temporal behavior of the weathering products at different immersion depths was quantified, and the consequent acid water was introduced to concrete blocks (simulating concrete structures) to examine the changes in the chemical and mechanical properties of the concrete.

## 2. Materials and Methods

### 2.1. Black Shale Samples

In this study, black shale samples from the Cambrian Qingxi Formation were sampled from Sanjiang County, Guangxi, China. The area has a humid tropical climate, and the black shale is abundant in carbon and pyrite, with a scarcity of fossils. Rock samples were taken from a depth of 1 m from the outcrop surface, following the procedure described in Liao et al. [7]. The rock samples were then covered by plastic wrap and transported to the lab. In the lab, the fresh surface of the black shale sample was cut out, and they were processed into cylindrical specimens (50 mm in diameter and 100 mm in height), using a core drilling machine (Hualian ZS-100, Taizhou, China). The specimens were drilled perpendicular to the bedding plane of the shale layer to ensure the same characteristics and entirety. Specimens with visual defects and larger discreteness were discarded. Mineralogical analysis of the black shale samples was conducted with X-ray diffraction (XRD; Rigaku Geigerflex RAD-IIB, Tokyo, Japan) of randomly oriented powder mounts, using CuKα radiation at 20 kV and 50 mA. The scan was conducted from 2θ = 3° to 40° with 2 s dwell time. The results showed that the major mineral phases of the black shale samples were quartz (SiO_2_), illite ((K,H_3_O)(Al,Mg,Fe)_2_(Si,Al)_4_O_10_[(OH)_2_,(H_2_O)]), and pyrite (FeS_2_).

### 2.2. Laboratory Black Shale-Concrete Immersion Tests

**Figure 1:**
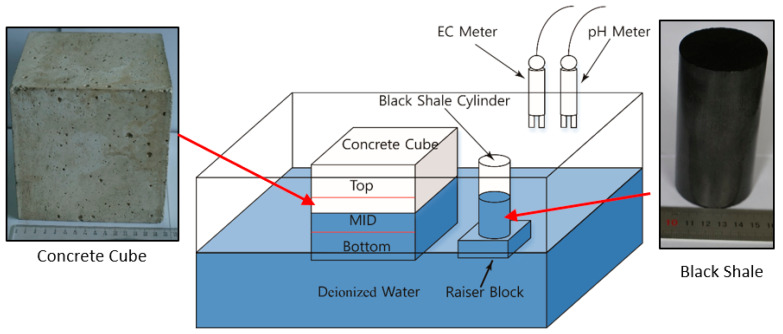
Schematic view of the setup of the laboratory immersion tests.

To evaluate the effect of the acidic discharge produced by black shale on the concrete, laboratory black shale-concrete immersion tests were set up to mimic in situ conditions. In this study, the cylindrical black shale specimens were partially immersed in deionized (DI) water at different immersion heights (30 mm and 50 mm or 30% and 50% immersed), which was designed to simulate the water-rock interactions under partially saturated conditions (Figure 1). The black shale specimens were immersed in a 300 mm (length) × 200 mm (width) × 200 mm (height) mm acrylic tank with DI water filled to 75 mm. The original pH and electrical conductivity (EC) of the DI water were 7.1 and 1.8 mS/m. The top of the tank was vented to the air, and the tests were controlled at a room temperature of 20 ± 1 °C and relative humidity of 30%. An acrylic raiser block was used to control the immersion depths of the black shale to be either 30 or 50 mm.

At the same time, a concrete cube (150 mm × 150 mm × 150 mm) was placed in the acrylic tank to evaluate the effect of acid discharge from the black shale on the physical and chemical properties of the concrete. During the immersion tests, half of the concrete cube (75 mm) was immersed in the DI water. The concrete cube was created by mixing 1060 kg/m^3^ of coarse aggregate (5–25 mm continuous-grade crushed stone), 750 kg/m^3^ of fine aggregate (natural river sand), 7.5 kg/m^3^ of polycarboxylate superplasticizer, and 410 kg/m^3^ of cement with water (water/cement ratio = 0.45) before it was cured for 28 days in a room with 100% humidity. The initial mineralogy analysis of the fresh concrete specimen showed that the concrete cubes mainly consisted of quartz, albite (Na(AlSi_3_O_8_)), slaked lime (Ca(OH)_2_), and Magnesium silicate hydroxide (Mg_3_Si_4_O_10_(OH)_2_).

The testing program of laboratory immersion tests is shown in Table 1, and the control groups (No.1 and No.6) were tested under scenarios without black shale and concrete, respectively. Tests No.2–No.5 had the concrete and black shale specimens. Tests No.2 and No.3 had an immersion depth of 30 mm for black shale, while No.4 and No.5 had an immersion depth of 50 mm for black shale. Tests No.3 and No.5 were duplicate tests of No.2 and No.4, respectively. Before immersion, the rock and concrete samples were cleansed by ultrasonic cleaning for 40 min to remove impurities. Online EC and pH meters were used for monitoring the chemical parameters of the water every 0.5 days for the first 15 days and then every day until a 40-day testing period was reached.

### 2.3. Water Chemistry Analysis

Water samples from the acrylic tank were taken after the 40-day testing period. The water samples were purified after centrifuging at 4000 rpm for 20 min, and the supernatant was then filtered through 0.45 μm filters and transferred to a 15-mL polypropylene centrifuge tube for chemical analysis. The bulk chemical parameters, including pH and EC, were analyzed using a benchtop pH and conductivity meter (Orion Star A125, Thermo Scientific, Waltham, MA, USA). Major elements, including aluminum (Al), iron (Fe), magnesium (Mg), calcium (Ca), sodium (Na), silicon (Si), and potassium (K), were analyzed by inductively coupled plasma optical emission spectrometry (ICP-OES, Perkin Elmer Optima 5300 V, Waltham, MA, USA). The major anions, including chloride (Cl^−^) and sulphate (SO_4_^2−^), were analyzed by ion chromatography (IC, Metrohm 792 Basic IC, Herisau, Switzerland).

### 2.4. Ultrasonic Speed Test

**Figure 2:**
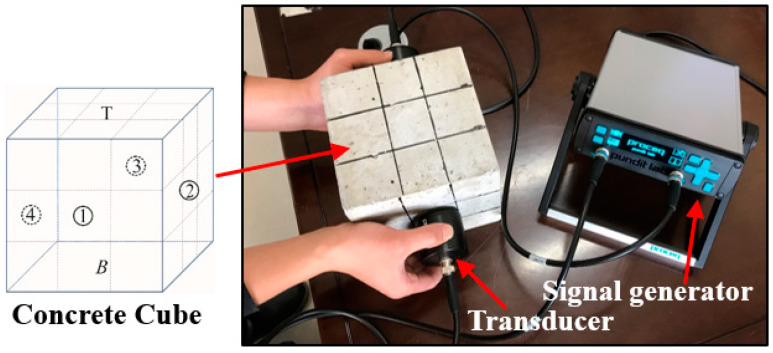
The setup of the ultrasonic speed test. The different surfaces of concrete are denoted as ①, ②, ③, ④, T, and B. T and B represent the top and bottom surfaces. Each surface of the concrete was marked into 3 × 3 partitions and then measured with the ultrasonic test equipment.

In this study, ultrasonic speed tests were performed to check the quality and continuity of the concrete before and after contact with the acid discharge from the black shale. The ultrasonic speed tests have been widely used for the inspection of concrete structures and presented high sensitivity and reliability to the interface between concrete and cracking [21,22]. The tests were conducted using ultrasonic test equipment (Proceq Pundit Lab+, Schwerzenbach, Switzerland), following ASTM E494-15. The test setup included (Figure 2): (1) a pair of standard transducers for emitting and receiving signals (natural frequency of 54 kHz); (2) a unit for signal generation, acquisition, and preliminary analysis; (3) a computer system for data storage and signal processing; and (4) dedicated software (Proceq Pundit Link, Schwerzenbach, Switzerland) that unlocks the full capabilities of the ultrasonic test system. The energizing signal is a square wave with an input voltage of 500 V. This high voltage allows the signal to be received even under a high attenuation scenario. Each lateral face of the concrete specimens was marked into a 3 × 3 partition, and the changes of the ultrasonic speed were determined at the top (non-immersed), middle (partially immersed), and bottom (fully immersed) sections of the concrete. For each section, three measurements were performed and the mean value (with the standard deviation) was reported.

### 2.5. Uniaxial Compressive Strength (UCS) Test

The uniaxial compressive strength (UCS) of each test specimen (black shale and concrete) after the laboratory immersion tests was evaluated by an electro-hydraulic servo rock test system (MTS815.03 MTS L. C., Eden Prairie, MN, USA). The UCS test is considered to be time-efficient, low-cost, and easy to operate. The length to diameter ratio (L/D) of the cylindrical black shale was set to be 2 (i.e., D = 50 mm and L = 100 mm), and the L/D of the cubic concrete was set to be 1 (i.e., L = D = 150 mm), which is consistent with the requirement in ASTM C39/C39M-12. During the tests, the loading displacement was set to be 0.1 mm/min until the specimen failed and the maximum normal stress could reach up to 100 MPa. Stress and strain results were recorded through an electronic system that has 1% accuracy.

## 3. Results and Discussions

### 3.1. Temporal Behavior of the Water Chemistry

#### 3.1.1. pH of the Water of the Immersion Tests

**Figure 3:**
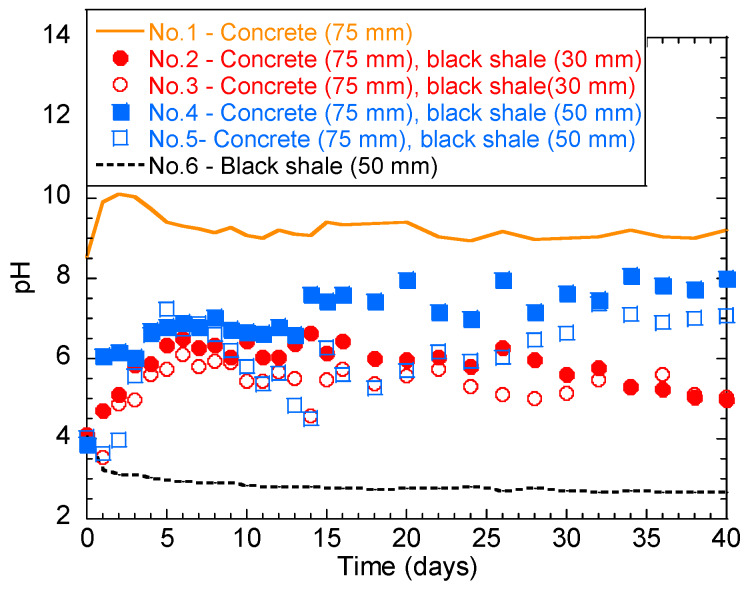
Temporal behavior of the solution pH during the laboratory immersion tests.

The temporal behavior of the solution pH in the laboratory immersion test is shown in Figure 3. Generally, concrete and black shale created alkaline (Figure 3 solid line) and acidic environments (Figure 3 dashed line), respectively. The pH of group No.1 (only concrete) was alkaline from 8.6 to 10.0 for the first 3 days, indicating the dissolution of the alkaline phase into the solution. The pH then slightly decreased and stabilized in the range of 9.0–9.5 after 5 days. The pH of No.6 (black shale only) showed a pH range of 2.5–4.0, indicating the oxidation of sulphide minerals and the generation of acid.

However, a different temporal behavior was observed for tests with both black shale and concrete specimens. The initial pH values of No.2 and No.3 (concrete immersion depth = 75 mm; black shale immersion depth = 30 mm) were both 4.1. These pH values increased within 6 days and then fluctuated in the weak acid range (5.0–6.5). The deeper immersion of black shale induced slightly lower water pH during the early stage of weathering. The initial pH values of No.4 and No.5 (concrete immersion depth = 75 mm; black shale immersion depth = 50 mm) were 3.9 and 4.1, respectively. However, a similar change of pH was observed within the first 6 days as that of No.2 and No.3, where the pH values increased within 6 days and then fluctuated between 6.5 and 7.5. This change of pH could be due to the balance between the acid-producing (pyrite oxidation) and alkali-producing (concrete dissolution) reactions. In general, the pH values of groups No.2 and No.3 were lower than those of groups No.4 and No.5, indicating that a lower immersion depth generates a lower overall solution pH.

#### 3.1.2. Electrical Conductivity and Elemental Concentrations in Water

**Figure 4:**
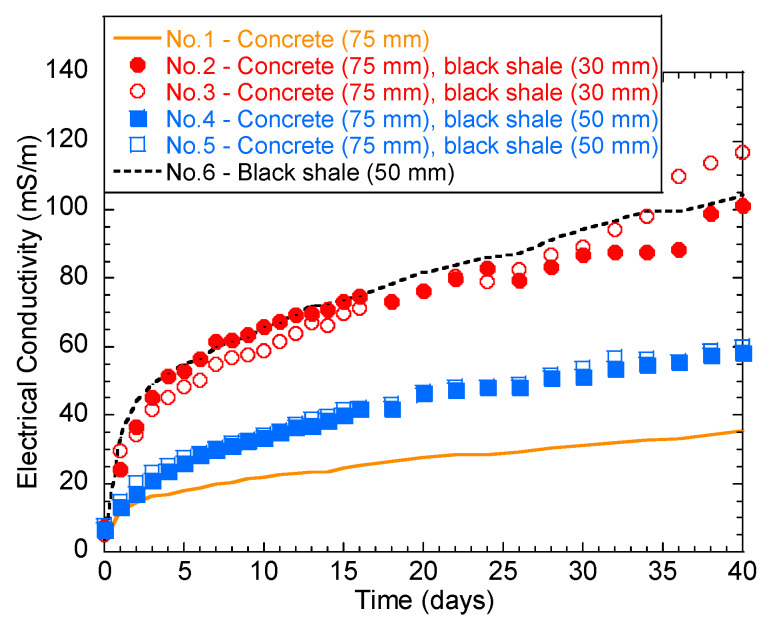
Temporal behavior of the solution electrical conductivity (EC) during the laboratory immersion tests.

**Figure 5:**
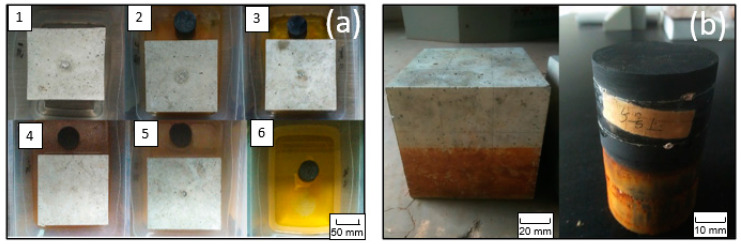
The visible presence of oxidation products (**a**) in the water and (**b**) on the surface of the concrete and black shale.

The temporal behavior of solution EC is shown in Figure 4. Generally, the EC of the solution increased rapidly over the first 2 days. The increase rate then slowed down until the 40th day. Tests No.2, 3, and 6 had the highest final EC (101.3–118.6 mS/m), indicating a stronger ionic concentration in the immersion solution than the other groups. In particular, the higher water EC of No.6 (black shale only) was majorly contributed to Fe (115.2 mg/L) and SO_4_^2−^ (1022.9 mg/L), which are the major oxidation products of pyrite (Table 2). Additionally, the color of all the immersed solutions turned yellow after immersion, except for group No.1, indicating the presence of ferric ion (Fe^3+^) (Figure 5a). The oxidation products were also visible on the surface of the concrete and black shale below the immersion level (Figure 5b). On the contrary, concrete (No.1) showed the lowest EC during the entire testing period, and the final EC on the 40th day was 35.4 mS/m. This result indicated that the concrete was relatively stable during immersion and the dissolution of major constituents from the concrete was relatively slow.

Increased release of Ca and K was evident when acid discharge from the black shale attacked the concrete. The concentration of Ca increased from 2.8 mg/L (No.1) to 20.0–436.6 mg/L (No.2–5) and the concentration of K increased from 97.6 mg/L (No.1) to 127.7–314.6 mg/L (No.2–5). These elements come from the dissolution of the major constituents of the concrete (e.g., Ca(OH)_2_ and KOH), which can be easily neutralized and dissolved by acid attack. The lower immersion depth (30 mm, No.2 and 3) produced a higher abundance of oxidation products, such as Fe and SO_4_^2−^, than tests with a higher immersion depth (50 mm, No.4 and 5). This phenomenon is consistent with the pH of the water, where the pH is higher with a deeper immersion depth. The reason for this is likely due to the higher exposed surface to the atmosphere at a lower immersion depth where oxygen is more easily obtained. The higher pH of No.4 and 5 also induced a higher potential for mineral precipitation. The water chemical parameters (in Table 2) were input into the geochemical model Phreeqc (Parkhurst and Appelo [23]) to calculate the saturation index (SI) of the minerals. The results indicate that goethite (FeOOH, SI = 2.5–8.2) and tricalcium aluminate (3CaO·Al_2_O_3_·6H_2_O, SI = 6.6–10.7 at No.4 and 5) were oversaturated in the water after the laboratory weathering tests (Table 3). 3CaO·Al_2_O_3_·6H_2_O is a typical hydration product of the concrete (Equation (1)), while FeOOH is the precipitation by the excessive ferric ions in water (Equation (2)).

2Al(OH)_3_ + 3CaO + 3H_2_O → 3CaO·Al_2_O_3_·6H_2_O (1)

Fe^3+^ + 2H_2_O → FeOOH(s) + 3H^+^ (2)

Ca^2+^ + SO_4_^2−^ → CaSO_4_ (s) (3)

CaSO_4_ (s) + 2H_2_O → CaSO_4_·2H_2_O (s) (4)

This precipitation of FeOOH was driven by the change in water pH, where the concrete was alkali and brought in hydroxide to the immersion water. Additionally, the presence of the oxidation product SO_4_^2−^ also brought the potential for precipitation of anhydrite (CaSO_4_) and gypsum Equations (3) and (4), where the saturation index was close to 0 (−0.1 to −1.7 for CaSO_4_ and −0.3 to −2.0 for CaSO_4_·2H_2_O). The presence of these minerals may induce swelling and cracking if precipitation occurs in the pore structure of the concrete due to the volume change of these minerals [18,19].

In general, the effect of immersion depth on the weathering of black shale was evident. A lower immersion depth represented a lesser contact surface with water. However, in this study, a lower immersion depth of black shale led to a higher degree of pyrite oxidation. The concentration of weathering products, such as Fe and SO_4_^2−^, in the immersion solution was relatively higher in the tests with lower immersion depth. Therefore, the saturated zone may not be very critical for pyrite oxidation compared with the capillary zone. Benavente et al. [24] found that the capillary zone of sedimentary rock can be a critical area for the water−rock interaction and is able to transport the products of the interaction. In this study, the weathering products were likely to have been generated and transported through the capillary zone to the immersion solution.

### 3.2. The Quality and Continuity of the Concrete before and after the Immersion Tests

**Figure 6:**
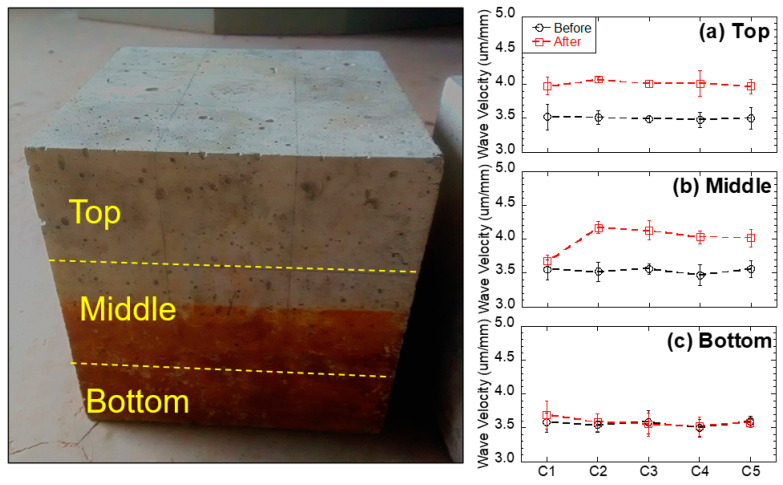
Measured ultrasonic velocity of concrete samples before and after the experiment: (**a**) top, (**b**) middle, and (**c**) bottom layers.

**Figure 7:**
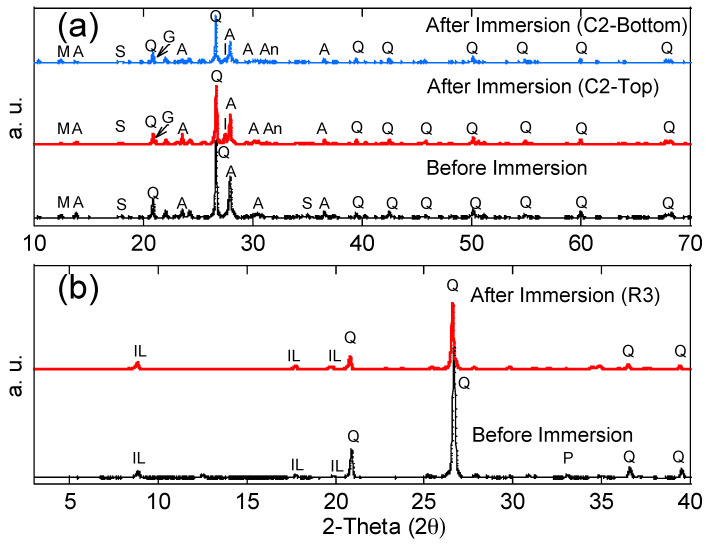
X-ray diffraction patterns of representative (**a**) concrete and (**b**) black shale specimens before and after immersion tests. Albite (A), Anhydrite (An), goethite (G), iron sulfate hydroxide (I), illite (IL), magnesium silicate hydroxide (M), pyrite (P), quartz (Q), and slaked lime (S).

Ultrasonic velocity increases with the density and continuity of the sample. Therefore, in this study, higher ultrasonic velocity revealed a better quality and continuity (less voids) of the concrete. The ultrasonic velocity of each concrete specimen before and after the immersion tests is shown in Figure 6, and average values with the standard deviation are reported. The standard deviation was less than 5% of all average values for all the tests. Before the immersion tests, the ultrasonic velocity of each concrete specimen was in the range of 3.5–3.6 um/mm, indicating that the concrete was uniform. After the immersion tests, the ultrasonic velocity of the top and middle layers had increased (from 3.7 to 4.1 um/mm), indicating the enhancement of the concrete quality (less voids) in these layers. This phenomenon is likely due to two mechanisms: (1) For the non-immersed (top) layer, since all testing groups had no contact with acid water, but increased ultrasonic velocity, the reason is most likely due to the additional hydration from capillary suction, which enhanced the strength of the concrete (e.g., C1 group without black shale); (2) for the partially immersed (middle) layer, since only groups with black shale presented increases in ultrasonic velocity, the precipitation generated in the capillary zone may have filled the voids of the concrete and increased the density and continuity of the material. The latter mechanism was further proved by the mineralogy analysis using XRD. The minerals of concrete before and after the immersion are reported in Figure 7a, and representative concrete specimens, i.e., C2 and C4, were selected and reported. The results show that goethite, iron sulfate hydroxide (Fe(OH)SO_4_), and anhydrite (CaSO_4_) appeared in the mineral phase after the immersion, indicating the precipitation of these minerals in the void spaces of the concrete. The precipitation occurred in both the immersed and non-immersed zones, showing that the capillary zone may have a strong effect on the mass transfer under partially immersed conditions.

However, no obvious change in ultrasonic velocity in the bottom layer was evident in general. The bottom layer presented a slightly lower ultrasonic velocity under acid water (3.6 µm/mm of C2 to C5) compared to that of concrete without acid water (3.7 µm/mm of C1). This phenomenon indicates that immersion in acid water would have a slightly negative impact on the quality of the concrete. This layer was immersed in the water, and the concrete underwent strong mineral dissolution. Although the precipitation of anhydrite, iron sulfate hydroxide, and goethite were evident in this layer (Figure 7a), the dissolution of minerals is dominant in the coupling effect of dissolution, precipitation, and hydration on the concrete and results in a reduction in the quality and continuity of the concrete after the immersion.

### 3.3. Compressive Strength of the Concrete and Black Shale before and after the Immersion Tests

**Figure 8:**
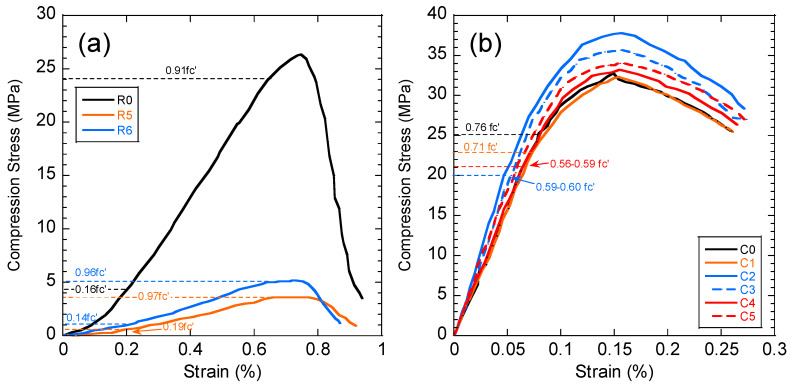
Stress-strain curve of (**a**) black shale and (**b**) concrete specimens after the immersion tests. C0 and R0 denote the concrete and black shale specimens before the immersion tests.

**Figure 9:**
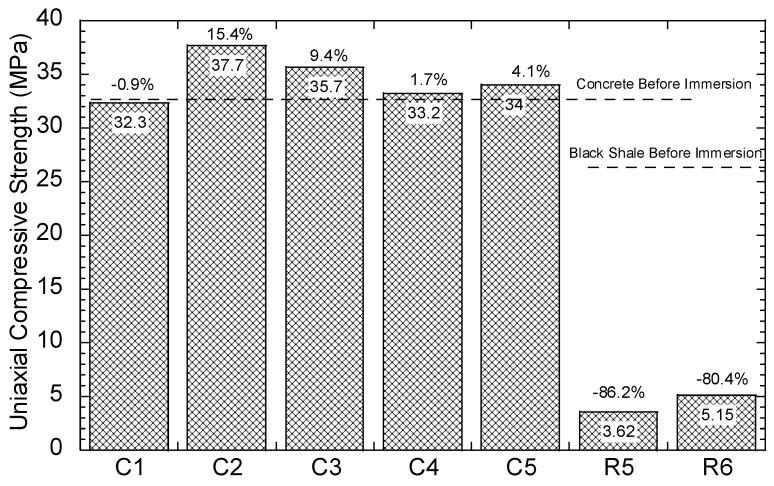
Uniaxial compressive strength of the concrete and rock samples.

The compressive strength of the concrete and black shale samples was examined before and after the immersion tests. Black shale specimens from R2, R3, and R4 could not be obtained due to the inordinate fractures and cracks. Therefore, only the relatively integrated black shale specimens (R5 and R6) and concrete specimens were tested in this study. The strains to reach the maximum compressive strength (fc’) were similar before (0.7%) and after (0.6%) the immersion, and the elastic ranges of the black shale were also identical for all immersion conditions (Figure 8a). The elastic range of the black shale generally appeared between the strain of 0.2% (at 0.14–0.16fc’) to 0.6% (at 0.91–0.97fc’). Destruction of the black shale specimens (R5 and R6) was observed after the immersion test. The maximum compressive strength of R5 and R6 decreased by 80.4–86.2% compared to that of the black shale before the immersion test (R0), which indicates that a strong weathering and dissolution occurred in the black shale (Figure 9). Additionally, the mineralogy analysis by XRD proved that the dissolution of minerals during the immersion tests, especially the pyrite peak, disappeared after the immersion period (Figure 7b). The dissolution of minerals also affected the strain-stress behavior of the black shale. The elastic modulus decreased dramatically from 4.5 GPa to 0.7 GPa, indicating a significant loss of stiffness in the black shale specimens.

The concrete had a slight reduction in compressive strength (0.9%, C1) after immersion in DI water for 40 days but had significantly increased compressive strength in acid water (1.7–15.4%) (Figure 9). Similar observations were found by Zhang et al. [25], using concrete specimens under sulphate attack with full immersion and wet-dry cycles. They proposed a three-stage model to demonstrate the change in compressive strength of concrete under sulphate attack: (1) the early enhancement stage, (2) the middle incubation stage, and (3) the later degeneration stage. In this study, the testing results revealed an early stage of the concrete under the contact with the acid water, which is consistent with the results found by Zhang et al. [25]. The enhancement stage occurs due to the filling effect of sulphate reaction products, such as gypsum and ettringite, in the voids of the concrete [23]. In this study, precipitations, such as goethite and anhydrate, produced by the interaction between concrete and acid water, were likely to fill the pore and fissures in the concrete and induce a similar enhancement effect. Additionally, the SI of gypsum was close to saturation in the water but highly likely to be oversaturated in the pore fluid of the concrete and precipitates.

The stress-strain curves of concrete presented a similar pattern before and after the immersion tests, and the strains (0.15–0.16%) to reach the maximum compressive strength were identical (Figure 8b). The immersion in DI water (C1) had no obvious impact on the stress-strain behavior of the concrete, while the elastic modulus increased from 29.4 GPa (C0) to 29.9–30.4 GPa (C2–C5) after the immersion in acid discharge, indicating that the concrete became stiffer after immersions. However, the elastic range of the concrete strongly decreased from 0.76fc’(C0) to less than 0.60fc’ (C2–C5) after the immersion in the acid discharge (dash line in Figure 8b). Combined with the mineralogy and water chemistry analysis of the current study, two mechanisms that affect the mechanical behaviors of the concrete could be found: (1) the strain-stress behavior of the concrete (shortening the elastic zone) by dissolving the alkali (e.g., Ca(OH)_2_ and KOH); (2) form precipitations of minerals and filling the voids. The latter mechanism presents a positive impact on the compression strength of the concrete in short-term tests, based on the findings of the current study. However, long-term tests are necessary to evaluate the long-term change of the compressive strength of the concrete.

## 4. Conclusions

This study evaluated the effect of the acid discharge from the weathering of black shale on the strength of concrete. Immersion tests were conducted at different immersion depths of the black shale to evaluate the oxidation conduction under partially saturated conditions. Concrete specimens were placed in the same water tank and directly contacted the acid discharge from the black shale. The oxidation products in the water and precipitation were quantified and calculated through water chemistry and mineral composition analysis during and after the immersion tests. The ultrasonic wave velocity and uniaxial compressive strength were obtained before and after the immersion by testing to evaluate the quality and strength of the black shale and concrete specimens.

Based on the results of this study, the following conclusions can be drawn from this research:

- Under the partially saturated condition, the oxidation of sulphide minerals in the black shale, i.e., pyrite in this study, depended on the immersion depth. Lower immersion depth of the black shale led to a higher degree of oxidation with lower pH, higher EC, and more abundant weathering products, such as SO_4_^2−^, and Fe, in the immersion solution. This observation was likely due to the oxidation and mass transfer in the capillary zone in black shale. Additionally, the oxidation and dissolution of minerals reduced the compressive strength of black shale.
- The acid discharge from the black shale showed a negative impact on the quality and entirety of the concrete in the immersed zone, but presented a positive impact on the non-immersed zone. The ultrasonic velocity of the concrete decreased in the immersed zone, which was due to the dissolution-induced reduction of concrete quality and entirety. However, the ultrasonic velocity of the concrete increased in the non-immersed zone. This phenomenon attributes to the mineral precipitation and hydration that occurred in the non-immersed or partially-immersed zones.
- In the testing period of the current study, the compressive strength of the concrete enhanced after contacting the acid discharge from the black shale. This phenomenon is attributed to the filling of the voids by the precipitations, such as goethite and anhydrite. However, a long-term test is needed in the future to evaluate the long-term impact of acid discharge on the compressive strength of the concrete.

## Figures and Tables

**Table 1:** Testing program of the laboratory immersion tests in this study.

Group No.	Specimens	Immersion Depth	
		Concrete	Black Shale
No.1	Concrete (C1 *)	75 mm	–
No.2	Concrete (C2) and black shale (R2 **)	75 mm	30 mm
No.3 ***	Concrete (C3) and black shale (R3)	75 mm	30 mm
No.4	Concrete (C4) and black shale (R4)	75 mm	50 mm
No.5	Concrete (C5) and black shale (R5)	75 mm	50 mm
No.6	Black shale (R6)		50 mm

* C represents concrete specimens and C1 denotes the concrete specimen that was not immersed in water. ** R represents rock specimens of black shale and R0 denotes the black shale specimen that was not immersed in water. *** No.3 and No.5 are the duplicate groups of No.2 and No.4, respectively.

**Table 2:** Water chemistry of the immersion solution after the 40-day laboratory immersion tests.

Content	No.1	No.2	No.3	No.4	No.5	No.6
pH	9.2	5.0	5.1	8.0	7.1	2.7
EC (mS/m)	101.3	116.8	58.35	60.1	104.1	101.3
Na (mg/L)	32.7	38.9	41.6	39.4	46.3	1.3
K (mg/L)	97.6	244.5	314.6	127.7	140.4	2.3
Ca(mg/L)	2.8	278.3	436.6	20.0	79.5	1.5
Mg (mg/L)	n.d. *	8.2	11.8	0.6	1.6	3.3
Al (mg/L)	n.d.	2.1	0.6	0.2	1.8	25.7
Fe (mg/L)	n.d.	13.0	15.5	0.2	2.4	115.2
Si (mg/L)	1.9	0.6	0.7	1.7	1.4	0.1
Cl^−^ (mg/L)	2.9	4.3	1.0	2.8	3.3	0.9
SO_4_^2−^ (mg/L)	8.2	1121.9	1625.2	250.2	369.1	1022.9

* non-detectable (n.d.).

**Table 3:** The calculated saturation index of minerals in the water of the laboratory immersion tests.

Content	No.1	No.2	No.3	No.4	No.5	No.6
Illite	0.0	−5.3	−5.0	0.6	3.8	−25.5
FeOOH	0.0	2.5	3.1	8.0	8.2	−3.4
SiO_2_(a)	−1.8	−2.3	−2.2	−1.8	−1.9	−3.2
CaSO_4_·2H_2_O	−3.8	−0.4	−0.1	−1.7	−1.1	−2.6
CaSO_4_	−4.1	−0.6	−0.3	−2.0	−1.3	−2.8
Ca(OH)_2_	−9.6	−15.9	−15.4	−10.4	−12.1	−22.7
3CaO·Al_2_O_3_·6H_2_O	0.0	−9.8	−8.7	6.6	10.7	−53.8
3CaO·Al_2_O_3_·3CaSO_4_·32H_2_O	0.0	−13.3	−12.7	−5.1	−3.1	−35.3
